# Genetic findings in sport-related concussions: potential for individualized medicine?

**DOI:** 10.2217/cnc-2016-0020

**Published:** 2017-01-24

**Authors:** Jane McDevitt, Evgeny Krynetskiy

**Affiliations:** 1 East Stroudsburg University, Athletic Training Department, East Stroudsburg, PA 18301, USA; 2 Temple University School of Pharmacy, Pharmaceutical Sciences Department, Philadelphia, PA 19140, USA

**Keywords:** MTBI, SNPs, sports medicine, TBI

## Abstract

Concussion is a traumatic transient disturbance of the brain. In sport, the initial time and severity of concussion is known giving an opportunity for subsequent analysis. Variability in susceptibility and recovery between individual athletes depends, among other parameters, on genetic factors. The genes-encoding polypeptides that determine incidence, severity and prognosis for concussion are the primary candidates for genetic analysis. Genetic polymorphisms in the genes contributing to plasticity and repair (*APOE*), synaptic connectivity (*GRIN2A*), calcium influx (*CACNA1E*), uptake and deposit of glutamate (*SLC17A7*) are potential biomarkers of concussion incidence and recovery rate. With catalogued genetic variants, prospective genotyping of athletes at the beginning of their career will allow medical professionals to improve concussion management and return-to-play decisions.

## From mechanics to genetics & backwards

Concussion is defined as a traumatically induced, transient disturbance of the brain brought on by complex pathophysiological processes [1]. Concussions most frequently occur following an event that involves an acceleration–deceleration mechanism, such as a blow to the head, or the head striking an object.

In sport-related activity, the exact time and severity of concussion can often be tracked, thereby providing the initial time of injury for subsequent analysis. Such analysis reveals significant variability in the immediate effects and recovery processes between individual athletes [2,3]. Concussion threshold has yet to be established because the accelerations required to cause injury are dependent on a number of intrinsic and extrinsic factors. Extrinsic factors include impact magnitude, direction and location [4]. Intrinsic factors include head position upon impact, number and severity of prior impacts, and other individual characteristics of the athlete, including the genetic profile [5].

The idea that genetic background is an important risk factor in an organism's stress response has been in development for several decades and is documented by multiple examples [6,7]. The discipline of pharmacogenomics uses genetic analysis to predict a physiological reaction to a chemical stimulus, usually a medication [8]. This approach is now widely recognized in personalized medicine. In sport-related concussions, the initial insult is a mechanical stress (i.e., neuronal stretch), where if large enough (i.e., stretch greater than 15% of the neuron's resting length) it will elicit a secondary biochemical response [9]. The immediate effect of mechanical stress on the neurons is membrane depolarization followed by deregulated release of neurotransmitters, as described below. Multiple proteins mediate the cellular response to this traumatic event, and their level and functions are determinants of physiological consequences of the impact. The genes encoding polypeptides that determine incidence, severity and prognosis for concussion are the primary candidates for genetic analysis.

## Pathomechanics of concussion

Concussive forces in athletes are evaluated with head accelerometer sensors to detect the magnitude, direction and location of head impacts [10]. Most concussion injuries occur when the neuron is stretched beyond the 15% range, which is yet another measurable parameter in brain injury models [11]. A patient can sustain a concussion with as little as to 60 g to as high as 160 g. Sagittal plane (front-to-back) impacts are usually less damaging than a coronal plane (side-to-side) movement, while rotational injuries had the worst outcomes. As the acceleration and deceleration forces increase, structural and/or chemical damage also increases. From a mechanical point of view, stress waves generated by the collision cause nervous tissue strain, with stronger impact inflicting the damage deeper within the brain [12].

Concussions may bring about neuropathological changes; these acute signs and symptoms (s/s) largely reflect functional disturbance. This type of injury could lead to an alteration in mental status accompanied by one or more of the following s/s: headache, nausea, vomiting, dizziness, balance problems, feeling ‘slowed down’, fatigue, difficulty sleeping, drowsiness, sensitivity to light or noise, loss of consciousness, blurred vision, difficulty remembering and difficulty concentrating [13,14]. Due to the fact that concussion results in functional disturbances rather than structural deformation, abnormalities resulting from a concussion are not detected on standard structural neuroimaging, such as on a MRI or CT [15].

A history of concussive injuries has been classified as having the strongest known association with increased concussion susceptibility, with two- to six-times increased risk of sustaining another concussion in athletes with a previous history of concussive injuries. Athletes with concussion history are also more likely to experience an increase in the amount of concussive s/s present from baseline, and those that had a history of loss of consciousness from concussion suffered from mild cognitive impairments later in life [16,17].

Athletes respond differently to concussions not only in the manifested s/s but also in number of days to recover. Approximately 80–90% of athletes are returned to play (RTP) within 7–10 days [18]. Importantly, there are athletes who manifest rapid recovery and are symptom free within 7 days. On the other hand, some athletes have prolonged recovery, lasting 21 or more days [19]. Professional football athletes were shown on average to RTP within 14 days, although 2% of this population took over 14 days to recover. In different studies, the prolonged recovery has been defined as concussion s/s persisting between 10 and 21 days, with no consensus-based duration [1,19–20].

Several risk factors for prolonged recovery have been identified, including specific populations, for example, children [15]; playing specific sports, for example, football; persistent concussive s/s, such as dizziness [19] and history of previous concussion [21]. Dizziness at the time of injury is the greatest indicator of a prolonged recovery time [19]. Important predictive factors are the number of previous concussions and severity of concussive s/s [22]. In general, specific concussion s/s (e.g., more cognitive or migraine symptoms) have been associated with extended concussion duration. In addition, headaches lasting longer than 60 h, three or more s/s at initial injury, and the presence of fatigue or fogginess were associated with an increase in recovery time [23].

## Molecular pathophysiology of concussion

The processes initiated by ionic misbalance following mechanical stress were mostly outlined by the end of the 20th century and have been summarized in several excellent reviews [9,24]. A mechanical insult resulting from acceleration or deceleration of the skull causes stress to the neurons and initiates a complex cascade of neurochemical events. If a neuron is strained 5–10% beyond its resting length, the stress leads to several short-lived (2–3 min) ionic imbalances, where an action potential will fail to fire. If the strain is 15–20% past its resting length, this structural change releases intracellular calcium (Ca^2+^) and causes an efflux of potassium (K^+^). Over 20% beyond the neuron's resting length results in axonatomy, or complete tear of the neuron [25].

After the physical integrity of cellular membranes has been compromised, the initial molecular event is membrane depolarization, followed by deregulated release of neurotransmitters, first of all the excitatory amino acid glutamate. Upon massive release of glutamate, glutamate-gated ionic channels jump to action by promoting Ca^2+^ release intracellularly, and facilitating efflux of K^+^ ions. The balance between extracellular and intracellular glutamate is critical for brain functions, and there is no extracellular metabolism of glutamate in the brain; therefore, it is glutamate transporters that are responsible for restoring the balance. Finally, Na^+^/K^+^ ATPase is activated in order to re-establish the ionic balance. All these events are facilitated by proteins, activity and levels of which may differ in individual athletes because of inherent genetic variants (see the next section).

**Figure 1:**
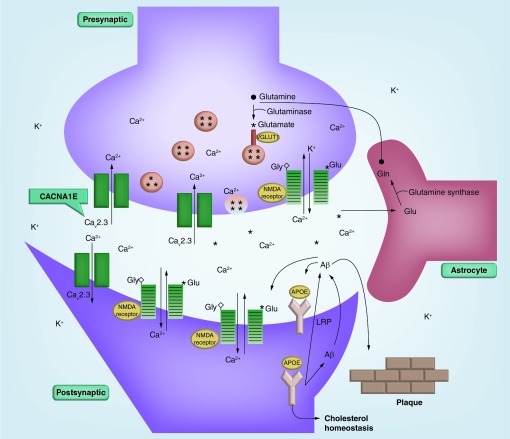
**The schematic of the neurometabolic cascade initiated by the mechanical disruption of cellular membranes and axonal stretching shows proteins responsible for plasticity and repair of neurons (APOE, LPR), ion channels (NMDA receptor, Cav2.3), and a neuromediator transporter (VGLUT1).** Persistent action potentials resulting from a neuron stretch release glutamate (shown as asterisks [*]) and other excitatory amino acids. Glutamate binds to the NR2 subunits within the NMDA receptors. This binding activates NMDA receptors, and initiates Ca^2+^ influx. Glutamate binding to NMDA receptor also leads to the efflux of K^+^ ions. Glutamate uptake into neurosecretory vesicles (shown as circles with asterisks) is facilitated by multiple glutamate transporter proteins including VGLUT1. In astrocytes (surrounding, supportive glial cells), glutamate is converted to inactive glutamine (•) by glutamine synthase, released to the extracellular compartment, and eventually reconverted to glutamate inside neurons in the course of the glutamine-glutamate cycle. Ca^2+^ enters the cells predominantly through the NMDA channels (striped rectangles), and voltage-activated Ca^2+^ channels (open rectangles), e.g., Cav2.3 encoded by *CACNA1E* gene. In response to injury, the synthesis of APOE is rapidly induced through the unique mechanism controlled by alternative splicing. The accumulating APOE participates in lipid transport and membrane repair. APOE binds to low-density lipoprotein receptor (LRP), and alters metabolism of amyloid-β (Aβ) peptides. Different APOE isoforms may affect intracellular trafficking of the Aβ peptide, and facilitate plaque buildup.

The simplified schematic of the neurometabolic cascade (Figure 1) outlines the major molecular components in the sequence of events initiated by the mechanical disruption of cellular membranes and axonal stretching (see Figure 1 for details).

In this review, we focus on genes from four functional groups. We selected genes which either were directly demonstrated to modulate concussion susceptibility/recovery or those for which reasonable evidence exists that link them to brain injury response. Specifically, we concentrate on genetic polymorphisms in the genes contributing to plasticity and repair (*APOE*), synaptic connectivity (*GRIN2A*), calcium influx (*CACNA1E*), uptake and deposit of glutamate (*SLC17A7*). Variability in the human genome is likely to modulate other concussion-related biochemical processes including hypoxia, inflammation and apoptotic pathways, which remain outside the scope of this review.

## Why do our genes govern concussion risk?

Variability between individual genomes of the same species is a common biological phenomenon. With thousands of individual human genomes available nowadays for comparative analysis, it is common knowledge that no two human genomes are identical. Each individual genome differs from the reference genome by about 3.5 million SNPs, including 400,000–600,000 new ones, about 1000 copy number variants exceeding 500 base pairs, other differences such as indels, inversions, and approximately 20,000–25,000 variants in coding sequences (with about 50% leading to changes in protein sequences). Of these gene variants, approximately 100 are loss-of-function variants, with about 20 genes completely inactivated [26]. The existence of millions of genetic variants also explains a continuum of physical characteristics that distinguish one human being from another. The most frequent type of genetic variability is an SNP. An SNP occurs when there is one nucleotide change at a certain position within the genome. This and other types of genetic variability may have profound effects on how the human body reacts and responds to stress, for example, head impact. Recent reviews summarize data on genetic factors contributing to incidence and outcomes of traumatic brain injury (TBI) [27,28].

The presence of an SNP in the DNA sequence can dramatically alter functions or the level of its protein product. For example, SNPs in the coding region of the gene can reduce catalytic activity, or decrease stability of the enzyme. A variation within the promoter region often reduces the amount of protein level, thereby abrogating its functions. Individual structural differences in neuronal proteins, such as channel proteins, could influence the way an individual's brain responds to an applied force, and may represent a predisposing or protective factor to concussions.

From a technical point of view, collection, purification and analysis of genomic DNA pose no difficulty even for a modest research facility [29]. Collecting biosamples (saliva or buccal swabs) requires neither medical intervention nor medical staff. DNA purification and genotyping reactions are highly automated procedures, with minimal involvement of personnel.

Besides genetic variants ‘hardwired’ into DNA sequence, other important biological mechanisms modulate gene expression (i.e., synthesis of gene products), such as epigenetic mechanisms. Understanding epigenetic mechanisms, for example, DNA methylation, histone modification and miRNA regulation is a prospective direction for molecular studies of concussion.

## Macromolecular components of the neurometabolic cascade

### Apolipoprotein E

One of the most studied genes associated with neurotrauma is *APOE*, which manifests genetic polymorphisms. The product of *APOE* is Apolipoprotein E (Apo E) glycoprotein, involved in redistribution of cholesterol during membrane synthesis, as well as neuritic growth, branching and repair. The role of Apo E in the pathophysiological response to brain injury and post-traumatic outcome is well documented (see [28] and references therein). Apo E exists in three isoforms, Apo E2, Apo E3 and Apo E4 encoded by three common alleles ϵ2, ϵ3 and ϵ4, respectively. The three allelic variants are distinguished by SNPs in exon 4 of the *APOE* gene. SNP rs7412C>T results in Arg158C is mutation and is present in alleles ϵ3 and ϵ4. SNP rs429358T>C results in Cis112Arg mutation and is present in allele ϵ4. The most common allele is ϵ3 (65–70% frequency), with two others being rarer variants (ϵ2, 5–10%, and ϵ4, 15–20%) [30]. Mutation at positions 158 of the Apo E2 polypeptide affects binding to the low density lipoprotein receptor (about 2% of normal activity), while mutation at position 112 of the Apo E4 polypeptide results in preferential binding of very low density lipoprotein particles and reduced Apo E4 stability. Structural and molecular modeling studies attributed these effects to altered interactions between two functional domains within the Apo E polypeptide chain [30,31].

Sport-related concussions are considered mild TBI, which are generally at the less severe end of the brain injury spectrum. The effects of Apo E allelic variants on TBI outcome are likely modulated by mechanical force, age or time post-TBI. Recent meta-analyses of studies investigating the role of Apo E4 on TBI indicate an association with an increased risk of poor outcome in pediatric carriers of at least one Apo E4 allele [32]. Such an association manifests differently depending on trauma severity: in mild TBI, Apo E4 association with post-TBI outcome was found noncontributory in 58.3% studies, while in severe TBI, the role of Apo E4 was hazardous in 63.6% studies. Another meta-analysis of six studies including a total of 358 cases of pediatric TBI revealed that at 6 months, there was over two-times higher odds of poor outcome following TBI in children with at least one APOE-ϵ4 allele, compared with children without ϵ4 allele [33]. In a meta-analysis of 12 studies on Apo E and functional outcome after TBI revealed an association with increased risk of unfavorable long-term functional outcome (≥6 months) [34].

While the relationship between APOE genetic variants and TBI has been established, the studies of APOE genotypes as concussion risk factors are still in progress. Several studies (e.g., see Table 1) demonstrated that the rare APOE variants are associated with greater risk of concussion, greater severity of symptoms, or poor outcomes following a concussion injury.

**Table 1.** **Genetic factors in concussion risk and outcome.**

**Pathway**	**Gene ID**	**Polypeptide**	**Polymorphism**	**Functional link of variant to brain pathophysiology**	**Ref.**
Nervous tissue healing	*APOE*	Apolipoprotein E	rs429358, rs7412	Deposition of Aβ	[35]
				Increased severity of neurological deficits in boxers	[36]
				Lower cognitive test scores in football athletes	[37]
				Poor outcome in young patients	[38]
				Lower Glasgow Outcome Scale scores at 6 months postinjury	[39]
				Poor outcome after TBI	[33]
			rs405509	Increased risk of Alzheimer's disease	[40]
				Association with poor TBI recovery in carriers of TT genotype	[41]
				Self-reported history of concussion	[42]
				Greater risk of concussion	[43]

Synaptic connectivity	*GRIN2A*	NR2A subunit of NMDA receptor	rs3219790	Higher risk of schizophrenia and bipolar disorder	[44,45]
				Increased risk of protracted concussion recovery	[46]

Calcium influx	*CACNA1A*	Calcium channel subunit	rs121908225	Brain swelling and coma after minor TBI	[47]
				Hemiplegic migraine, early seizures and cerebral edema after trivial head trauma	[48]
	*CACNA1E*	Calcium channel subunit	rs704326	Migraine	[49,50]

Uptake and deposit of glutamate	*SLC17A7*	Vesicle glutamate transporter VGLUT1	rs74174284	Eight patient-specific rare variants in 16 out of 376 patients with schizophrenia; no rare variants were found in 368 control subjects	[51]

Repair and plasticity	*BDNF*	Brain-derived neurotrophic factor	rs6562	Slow processing speed	[52]

Vascular response	*NOS3*	Nitric oxide synthase	rs2070744	Carriers of the C allele had lower cerebral blood flow values	[53]
				Higher risk of intracranial aneurysms in Asians	[54]
			rs1799983	TT genotype was associated with violent suicide attempts	[55]
			rs2070744	TT genotype increased expression of mRNA NOS3; CC carriers displayed lower NO metabolite level	[56]
				G894T and intron 4 VNTR risk-elevating genotypes in the same individual increased the risk of stroke	[57]
			TGT haplotype of rs1800783, rs1800779 and rs2070744	Carrying the TGT haplotype was associated with hypoxic-ischemic encephalopathy	[58]

Hypoxia response	*NGB*	Neuroglobin	rs3783988	TT carriers have 2.65-times greater likelihood of better outcomes on Glasgow Outcome Scale, the neurobehavioral rating scale-revised and the disability rating scale from 3 to 24 months	[59]

NMDA: N-methyl-D-aspartate; NO: Nitric oxide; NOS: Nitric oxide synthase; TBI: Traumatic brain injury; VNTR: Variable number of tandem repeat.

The molecular mechanisms leading to these effects are largely understood. While nonsynonymous SNPs (those changing amino-acid sequence of a polypeptide) can be detrimental for the protein activity, genetic polymorphisms outside the coding regions of the gene also affect protein synthesis. SNPs in the promoter region and intronic sequences often reduce gene expression or affect RNA maturation.

A study in 195 college athletes demonstrated that homozygous carriers of G(-219)T SNP in the *APOE* promoter region were at increased risk for having a history of concussion, but did not find any difference between ϵ2, ϵ3 and ϵ4 allele carriers [42]. The presence of all three alleles was significantly associated with history of concussion in collegiate athletes [43]. Within a small sample of 30 boxers, high-exposure boxers (12 or more professional matches) carrying ϵ4 allele had worse chronic brain injury scale scores. Within the same study, all boxers with severe impairment were found to carry at least one ϵ4 allele [36]. Taken together with clinical data on the risk of TBI in the carriers of *APOE* genetic variants, these data support the hypothesis that *APOE* genetic variants are a biomarker for concussion risk.

Despite pretty strong evidence suggesting the important role *APOE* allelic variants play in concussion incidence and recovery, conflicting results do not allow us to make formal confirmation of this hypothesis at this time. In a large cohort of 318 athletes, no important association between carrying the apolipoprotein ϵ4 allele and sustaining a concussion was detected [60]. The ultimate confirmation would require prospective, multicentered studies with sufficient power and carefully selected inclusion/exclusion criteria [61].

## Ionotropic glutamate N-methyl-D-aspartate receptor

The primary function of the N-methyl-D-aspartate (NMDA) receptor is to act as synaptic connectivity between two neurons as well as trigger postsynaptic potentials and dendritic spikes, where action potentials are formed. Mechanical stress activates NMDA receptors via overstimulation by increased glutamate concentration within the synaptic cleft. Ionotropic NMDA receptors are recognized as the major source of glutamate excitotoxicity (injury to the nerve due to excessive stimulation by glutamate) dependent on the influx of Ca^2+^ when glutamate binds to NMDA receptor.

NMDA receptors are composed of four subunits forming a ligand-gated cation (e.g., Ca^2+^) channel in which the NR1–NR2A heterodimer is the functional unit. The main NR2 subunits are NR2A and NR2B encoded by the *GRIN2A* and *GRIN2B* genes, respectively. *GRIN2A* is 3.4 kB in length located on chromosome 16 and contains multiple SNPs in the coding, intronic and promoter regions.

NMDA receptors are associated with learning and memory. Dysfunction of the NMDA receptors through deregulated trafficking [62], inherited variations in a subunit expression [63], variability in subunit expression level [64], treatment with anxiolytic NMDA receptor antagonists [65], or NMDA receptor enhancers [66,67] has been implicated in several cognitive brain diseases including dementia, Alzheimer's disease, depression, and schizophrenia [68].

Functional involvement of NMDA receptors in the concussion stress response is supported by several lines of evidence. An important role of polypeptide components of NMDA receptor was demonstrated in experiments with genetically manipulated mice. Less brain ischemia was detected in NR2A or NR2A/NR2B knockout mice, after they were subjected to focal cerebral ischemia by introducing a suture from left common carotid artery. The lack of NR2A is likely to alleviate glutamate excitotoxicity due to the decreased amount of blood volume, which could be explained by decreased NMDA channel activity [69]. The reduced functionality of the NMDA receptors results in less Ca^2+^ entering the cell [70].

Experiments with a fluid percussion in rats demonstrated the loss of NR2A subunits both bilaterally and ipsilateraly in the cerebral cortex following a head injury [71]. These NR2A levels were reduced for up to 4 days, while NR2B and NR1 subunits were not significantly affected. However, these changes were only seen on a protein level and not mRNA transcription levels, suggesting a nontranscriptional mechanism of regulation [72].

Clinical studies demonstrated associations between genetic polymorphisms in the NMDA subunit genes and response to pharmacological management of attention-deficit/hyperactivity disorder [73]. A separate study of the *GRIN1* and *GRIN2B* genetic variants demonstrated that individuals carrying polymorphisms have a reduced risk of Parkinson's disease, probably due to low activity of the NMDA channel [74].

The level of functional NMDA receptors is regulated post-translationally by Ca^2+^-activated proteases (calpains) [75]. Calpain activity increases significantly in experimental animals after TBI, resulting in proteolysis of glutamate receptors at the C-terminus of the glutamate receptor subunits [76,77]. In turn, calpains contribute to Ca^2+^-induced neuronal cell toxicity, and upon activation may have deleterious effects on neurons. Genetic and epigenetic regulation of calpain activity (either direct or through the regulatory network) is likely to influence the outcome of concussion; however, this hypothesis is still waiting to be tested [78–80].

Taken together, the above data support the premise that variability in NMDA receptor expression could be a risk factor for concussion outcome. Therefore, genetic variants of the genes coding for the components of NMDA receptor complex are attractive candidates for association with concussion incidence or recovery. One important type of genetic variability is the number of tandem repeats in the promoter region of *GRIN2A* gene (see the next section).

## *GRIN2A* simple tandem repeat polymorphism

Simple tandem repeats (STRs) are an important class of variable genetic elements. STRs have been found throughout the human genome, including coding sequences, introns and regulatory elements, for example, promoter regions. Analysis of STR distribution across the genome, expression experiments and association studies indicates that the variation in number of STR alters gene expression [81]. Therefore, STR is yet another genetic polymorphism that affects the gene expression pattern and may change phenotype of an organism [82].

**Figure 2.**
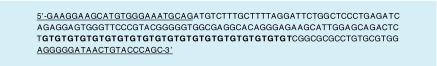
**A simple tandem repeat GT20 located in the promoter region of the *GRIN2A* gene (highlighted with bold font). The partial sequence of the promoter on chromosome 16p13.2 with coordinates (chr16:10277386-10277585, Feb. 2009 (GRCh37/hg19) Assembly) is shown.** Underlined regions were used for PCR primer design and subsequent sequence analysis [46].

The promoter region of *GRIN2A* gene is located on the short arm of chromosome 16 and contains dinucleotide repeats (GT)n (Figure 2). This locus manifested modest linkages for mood disorders including bipolar disorder and schizophrenia. Several compelling candidate genes are mapped to this area, due to their relevance to the ‘hypoglutametergic hypothesis’ for mood disorders [44,45]. In line with these reports, *GRIN2A* promoter polymorphisms were found to be linked with increased susceptibility to schizophrenia and bipolar disorder [44,83]. Concussion injuries are also associated with cognitive problems; therefore, this STR is hypothesized to have effect on concussion severity and recovery rate [46].

The variable number of STRs in the *GRIN2A* promoter region had been demonstrated to alter the expression level of *GRIN2A* [44,84]. The length of (GT)n repeat modulates *GRIN2A* mRNA expression level, with longer alleles (≥25 repeats) driving transcription of *GRIN2A* mRNA less efficiently than the shorter ones. In a study of 87 athletes suffering with a concussion, homozygous carriers of the longer alleles were found six-times more likely to recover in 60 or more days, compared with homozygous carriers of the shorter (<25 repeats) alleles [46].

With athletes categorized to one of two groups (prolonged recovery vs normal recovery), significant variation between the frequencies of longer alleles and shorter alleles was detected (p = 0.048), where the carriers of longer alleles were two-times more likely to be in the prolonged recovery group than those carrying shorter alleles. Moreover, homozygous carriers of longer alleles demonstrated a significant association with prolonged recovery when compared with homozygous carriers of shorter alleles (p = 0.0433). There was no association with other descriptive characteristics, such as age, sex, ethnicity, history and number of previous concussions, acute s/s at the time of injury, learning disability or migraine history.

Despite several limitations of this study including uneven distribution of sex and race between groups, and the time of initial assessment, the results of this study suggest that genetic polymorphism in *GRIN2A* promoter could be a useful predictive marker of athlete susceptibility to concussion.

## Vesicle glutamate transporters

The synaptic uptake of glutamate is facilitated by vesicular transporters (i.e., VGLUT1, VGLUT2 and VGLUT3) encoded by the solute carrier subfamily of genes located on chromosomes 19 (*SLC17A7*), 11 (*SLC17A6*) and 12 (*SLC17A8*), correspondingly. VGLUT1 performs synaptic uptake of glutamate and deposit into neurosecretory vesicles and is expressed in cerebrum, cerebellum and hippocampus. Downregulation of VGLUT1 vesicular transport production was shown to cause severe changes in neurological phenotype of experimental animals [85]. Knockout studies demonstrated reduction of vesicle pool size accompanied by residually high concentrations of glutamate within the synaptic cleft [86]. The resulting alterations in brain functions range from progressive phenotype expression to fatality. Though the VGluT1^-/-^ mice were born with no particular phenotype, after 3–4 weeks they manifested blindness, and were severely impaired in coordination, learning and memory [85]. VGluT1^+/-^ hemizygous mice showed deficit of behavioral flexibility when adapting to a new situation [87–90]. Glutamatergic nerve terminals in hemizygous mice demonstrated increased susceptibility to Aβ_1–42_-induced toxicity [91].

Another piece of evidence indicating that VGLUT activity could modulate synaptic efficacy came from the clinical studies of VGLUT expression in schizophrenic patients. VGLUT expression in the hippocampus and the dorsolateral prefrontal cortex was reduced in the brains of schizophrenic patients [92]. Resequencing and association studies in 376 schizophrenic Asian patients did not detect significant differences in allele or genotype distribution of common SNPs between patients and controls. Instead, eight rare variants were discovered in 16 patients and in none of the control group [51].

Several clinical studies suggest a link between the level of VGLUT and the response to pharmacological intervention. Chronic administration of antidepressant drugs increases the level of VGLUT1 in the cerebral cortex and hippocampus [93,94]. Response to treatment with selective serotonin reuptake inhibitors was significantly associated with the presence of SNP rs74174284:C>G in the 5′-region of the *SLC17A7* gene [95].

If the expression level of a protein responsible for reducing the amount of glutamate (e.g., VGLUT1) within the synaptic cleft is altered due to genetic polymorphism (e.g., SNP), this may affect an initial reaction to the concussive injury, and the recovery time. A study in 40 athletes demonstrated that the recovery time of the carriers of rs74174284:G allele in *SLC17A7* promoter was five-times more likely to exceed 20 or more days [96]. These results are in line with the hypothesis that low expression of *SLC17A7* encoding VGLUT1 probably reduces glutamate transport in the carriers of G allele [86]. If glutamate transport is reduced due to decreased vesicular uptake, concussive s/s could be exacerbated due to the disruption in neural transmission in this group of athletes. The excessive energy demand that is required to re-establish homeostasis may be too much of a strain on a developing brain in younger athletes, and requires a longer time period to recover [97,98].

## Voltage-activated Ca^2+^ channels

In response to membrane depolarization, voltage-activated Ca^2+^ channels mediate Ca^2+^ entry into a variety of cells including nerve, endocrine and muscle cells. Therefore, these proteins are key components of the neurometabolic cascade following mechanic stress and membrane depolarization. Calcium channel, voltage-dependent, R-type α1E subunit (CACNA1E) forms the R-type Ca_v_2.3 channel which in conjunction with the T, N, P and Q-type channels contributes to the overall calcium current in dendrites and activates the release of neurotransmitters. CACNA1E supports both presynaptic calcium influx that triggers neurotransmitter release, and postsynaptic calcium entry that helps shape the response downstream to that release. CACNA1E was shown to mediate a significant fraction of R-type Ca^2+^ currents, specifically within the hippocampus [99].

Experiments with knockout mice, as well as clinical data in several human populations demonstrated that genetic polymorphism of *CACNA1E* is likely to change neurologic biochemical pathways. Taken together with the role of Cav2.3 in neurometabolic cascade, these findings suggest *CACNA1E* as a possible modulator of postconcussion stress response, in other words, concussion severity and duration. Knockout mice with the disrupted *Cacna1e* gene are viable and available for phenotypic characterization. Alpha 1E-deficient mice (α1E^-/-^) exhibited behavioral defects, functional deficits in pain perception and altered response to cocaine [100]. Focal ischemia model in Ca_v_2.3-deficient mice suggested a protective role for Cav2.3 channel in ischemic neuronal injury [101]. SNP in the *CACNA1E* gene was associated with seizure susceptibility of Sprague-Dawley rats [102].

Human Ca_v_2.3 is encoded by *CACNA1E* gene located at locus 1q25–31 and contains 52 exons. The polypeptide product of *CACNA1E* is broadly expressed throughout the central and peripheral nervous system both pre- and postsynaptically. SNP analysis of the *CACNA1E* gene revealed association with several biochemical processes, such as myelinogenesis and pain perception [103].

While microsatellite analysis mapped the type 2 familial hemiplegic migraines susceptibility locus to the markers in the 1q31 and 1q23 loci, an association analysis of candidate genes failed to demonstrate a significant role for *CACNA1E* variants for familial hemiplegic migraines in 243 unrelated Caucasian suffering from migraine compared with 243 controls [49]. A weak association was found between rs704326:C>T in the open-reading frame of *CACNA1E* and bipolar disorder in the Asian population [104]. Moreover, genotyping analysis in about 9800 patients revealed an association with Type 2 diabetes [105]. Together with a well-known glycolytic component of neurometabolic cascade, these facts led us to hypothesize that *CACNA1E* could be a viable candidate gene associated with concussion risk. Although for the moment, no experimental data showing an association with concussion have been published, we speculate that *CACNA1E* could contribute to concussion incidence or outcome.

## Genetic tests in concussion studies

Genetic analysis of sport-related concussions aims to solve two problems. First, biomarkers indicative of certain risk factors may identify individuals vulnerable to concussion. This premise is based on the hypothesis that athletes carrying certain genotypes are more susceptible to concussion. Second, identification of biomarkers predictive of delayed recovery will help to guide the postconcussion management and to rationalize RTP decisions. In parallel to genomics approach to pharmacotherapy, two branches of concussion-related genetic studies are plausible. First, the search for biomarkers of risk factors with substantial predictive power. Second, the routine genotyping and processing of genetic information for the benefits of athletes.

The search for genetic biomarkers is usually performed in the course of genome-wide association studies (GWAS) by statistical analysis of many (in the range of thousands) genomes, and consumes significant resources in terms of money, time and computing power. Though GWAS are prone to false-positive associations, the advantage of GWAS studies is that most of the genes in the genome are interrogated for association with the phenotype, so that no preliminary hypothesis about genetic markers is needed.

An alternative approach is based on analysis of candidate genes, which have been preselected based on their functions. While much simpler and straightforward, such a study is inevitably limited to a subset of genes with known relationship to concussion. In practice, both strategies are applied in parallel. For example, with GWAS study used to generate a list of candidate genes, the candidate gene approach is practical for validation of biomarkers and population screening.

With the GWAS studies of concussion-related genes still in progress, several candidate gene studies support the prospects of genetics of concussion. The protein products of the genes selected for analysis participate in several biochemical pathways involved in concussion stress response, including repair and plasticity of neurons, transport and release of ions, inflammatory response, vascular and hypoxia response (Table 1). Depending on the functions and size of the gene, the number of SNPs to be interrogated in each gene may be in the range of hundreds, differing in functional effects and frequency in the population. The rational selection of SNPs for analysis is a prerequisite for the candidate gene approach. At this initial stage of genetics of concussion, one reasonable strategy is to test the genetic variants with known physiological effects in biochemical processes relevant for concussion stress. Above, we discussed several examples of such genes, with many more genes left unexplored.

With further characterization of the polymorphic ‘risk genes’ pertinent to sport-related concussion, a comprehensive list of genetic variants can be prepared, similar to a list of drug-metabolizing enzymes, transporters and drug targets already in existence for pharmacogenomic analysis. With catalogued genetic variants, a prospective genotyping of athletes at the beginning of their career would be quite affordable, and ready to guide RTP decision should it be needed. Such analysis is available for most genetic variants and needs to be performed only once in a lifetime.

To date, it still remains to be addressed whether athletes have any interest in genetic testing, or the extent to which the results would change the athlete's career decisions. To this end, a 38-item questionnaire to capture student athletes’ interest on genetic testing on the risk of poor recovery from concussion, and an increased risk for late onset Alzheimer's disease was created [106]. Eight hundred and forty-three (454 women, 389 men) National Collegiate Athletic Association athletes from 20 institutions completed the questionnaire. Close to half of the athletes reported they sustained a concussion (40%), and 15% recounted they had a difficult recovery. Nearly three quarters of athletes expressed some level of interest (55% possibly interested, 19% very interested) of genetic testing for an increased risk of poor recovery from concussion. Athletes who had experienced a difficult recovery were more likely to report being very interested in genetic testing. This was the first study to acknowledge that athletes are interested in their genetic risk and, more importantly, are willing to share this information. Athletes preferred meeting with the genetic counselor after and not prior to genetic testing, which suggests they may not truly understand genetic testing [106].

Athletes did not believe genetic testing could affect their involvement in sport, nor were the athletes concerned about the other possible ramifications of disclosing genetic testing results. However, this could change in real-life situations when detection of a ‘susceptibility gene’ could affect the chances of being drafted, getting contract or negotiating insurance. Similarly to genetic testing for diseases, athletes should be protected from misuse of genetic information, and confidentiality of tests should be supported by appropriate legislation. A public discussion among athletes, coaches and other members of the sports community might be appropriate to move us toward a balanced and rational approach to genetic testing and its use. The complex ethical problems associated with this question may, however, require a whole set of separate publications.

## Conclusion

Genetic testing to determine risk of injury or disease in sport is becoming a tool of utmost importance. While there is no way to exclude the risk of concussion in sport, an emerging knowledge of the human genome may help us to make more informed and rational decisions regarding susceptibility and recovery strategy for athletes.

## Future perspective

In 10 years, we will no doubt be better informed about the relationships between genotype (genetic profile) and phenotype (physical characteristics) in humans, specifically in athletes who might experience concussive trauma. Similar to other biomedical disciplines already embarked on personalized approach to medical intervention, an individual's human genome will reveal enough information for rational selection of predictive biomarkers relevant for concussion management. These biomarkers will allow us to prospectively identify athletes susceptible to concussive stress, and to select those who need additional recovery time before returning to play. Overall, the genetic analysis will help to improve quality of life and reduce the burden of TBI in athletes.

**Executive summary**

**Variability of the human genome**

- Multiple proteins mediate the cellular response to concussion, and their level and functions are determinants of physiological consequences of the impact.
- The timing and severity of concussion are often clinically observed and quite suitable for further correlative analysis using genetic tools.

**Pathomechanics of concussion**

- Athletes respond differently to concussions not only in the manifested signs and symptoms but also in number of days to recover, depending on a number of extrinsic and intrinsic factors.
- Extrinsic factors include impact magnitude, direction and location. Intrinsic factors include head position upon impact, number and severity of prior impacts, and other individual characteristics including the genetic profile of an athlete.

**Molecular pathophysiology of concussion**

- The initial molecular consequence of impact is the membrane depolarization followed by deregulated release of neurotransmitters.

**Macromolecular components of the neurometabolic cascade**

- Three classes of polypeptides are likely to be the most important candidates for genetic analysis that may determine severity and outcome of sport-related concussions.

**Genetic tests in concussion studies**

- Biomarkers may help to identify individuals vulnerable to concussion.
- Identification of biomarkers predictive of delayed recovery will help to guide the postconcussion management, and to rationalize return-to-play decision.
